# Diversity of Macro- and Micronutrients in the Seeds of Lentil Landraces

**DOI:** 10.1100/2012/710412

**Published:** 2012-09-10

**Authors:** Tolga Karaköy, Halil Erdem, Faheem S. Baloch, Faruk Toklu, Selim Eker, Benjamin Kilian, Hakan Özkan

**Affiliations:** ^1^Organic Agriculture Program, Vocational School of Sivas, Cumhuriyet University, Sivas, Turkey; ^2^Department of Soil Sciences, Faculty of Agriculture, Çukurova University, 01330 Adana, Turkey; ^3^Department of Field Crops, Faculty of Agriculture, Çukurova University, 01330 Adana, Turkey; ^4^Seed Science and Technology Program, Vocational School of Kozan, Çukurova University, Kozan, 01550 Adana, Turkey; ^5^Leibniz Institute of Plant Genetics and Crop Plant Research (IPK), Genebank/Genome Diversity, Corrensstraße 3, 06466 Gatersleben, Germany

## Abstract

Increasing the amount of bioavailable mineral elements in plant foods would help to improve the nutritional status of populations in developing countries. Legume seeds have the potential to provide many essential nutrients. It is important to have information on genetic variations among different lentil populations so that plant breeding programs can use new varieties in cross-breeding programs. The main objective of this study was to characterize the micro- and macronutrient concentrations of lentil landraces seeds collected from South-Eastern Turkey. We found impressive variation in the micro- and macroelement concentrations in 39 lentil landraces and 7 cultivars. We investigated the relationships of traits by correlation analysis and principal component analysis (PCA). The concentrations of several minerals, particularly Zn, were positively correlated with other minerals, suggesting that similar pathways or transporters control the uptake and transport of these minerals. Some genotypes had high mineral and protein content and potential to improve the nutritional value of cultivated lentil. Cross-breeding of numerous lentil landraces from Turkey with currently cultivated varieties could improve the levels of micro- and macronutrients of lentil and may contribute to the worldwide lentil quality breeding program.

## 1. Introduction

Lentil (*Lens culinaris *Medik.) is an ancient crop of classical Mediterranean civilization and continues to play an important role in the global human diet and modern agriculture. Lentil is one of the oldest dry legumes and was domesticated about 9000 years ago from the wild progenitor *Lens culinaris *subsp. *orientalis *(Boiss.), in an area that comprises modern day South-Eastern Turkey and an adjoining part of Syria [1]. Ferguson et al. [2] showed that South-Eastern Turkey/North-Western Syria is the primary center of diversity for *L. culinaris*.

Lentil is the fourth most important pulse (legume) crop in the world after bean (*Phaseolus vulgaris *L.), pea (*Pisum sativum *L.), and chickpea (*Cicer arietinum *L.). According to the Statistical Bureau of the Food and Agriculture Organization of the United Nations, lentil is currently cultivated on 4 million ha in warm temperate, subtropical, and tropical regions of more than 40 countries and is grown in all continents except Antarctica [3]. There are two biotypes of cultivated lentil: small seeded (*microsperma*) and large seeded (*macrosperma*). Microsperma lentils are widely grown in South-Eastern Turkey and provide an inexpensive source of protein. Lentils are commonly consumed throughout the Mediterranean and Middle East regions. They are also used to make nutritious and inexpensive soups that are popular in Northern Europe and North America. Turkey has its own version of red lentil soup locally known as “*Kırmızı Mercimek Çorbası*”, that is distinct from South Asian and North African dishes. The composition and nutritional quality of the lentil make it an important crop, especially in the developing world.

Mineral elements play important physiological roles in plants and in the human body. The human body requires more than 22 minerals that can be supplied by an appropriate diet [4], and the most important minerals are P, K, Ca, Mg, Fe, Zn, Cu, and Mn. Dietary deficiencies in mineral elements can have significant negative impacts, such as learning disabilities in children, increased morbidity and mortality, low worker productivity, and high healthcare costs. The most common micronutrient deficiencies are Fe, Zn, and I, but certain populations may also suffer from deficiencies in Mg, Ca, and Se. It has been estimated that nearly 3.7 billion people worldwide are Fe deficient (60%) and that 54% of these 3.7 billion people are severely deficient [5]. Zn deficiency ranks the 11th among the 20 most important nutritional deficiencies worldwide, and the 5th among the 10 most important deficiencies in developing countries [6]. Hotz et al. [7] reported that Zn deficiency affects about one-third of the world population and that its incidence ranges from 4% to 73% depending on the country. Micronutrient deficiencies mainly result from low concentrations in the daily diet. The concentrations of most minerals in most plant foods are not sufficient to meet daily dietary requirements when these foods are consumed in typical amounts. Hence there has been an interest in increasing the mineral concentrations of various seed crops. Although food supplements were traditionally used to treat mineral deficiencies, agricultural strategies for increasing micronutrient density in foods are now being assessed as sustainable and long-term solutions.

Micronutrient deficiencies are a significant problem in Turkey and in the Mediterranean region. Fe and Zn deficiencies are quite common, especially in school children and women, mainly due to the high proportion of monotonous cereal-based foods in typical Turkish diets. In recent years, Zn and Fe deficiencies have received particular attention in Turkey and the rest of the world [6]. Regions with Zn-deficient soils, such as India, Pakistan, China, Iran, and Turkey, are also regions where human Zn deficiency is most widespread [7, 8]. Eyüpoğlu et al. [9] reported that more than 50% of the land (14 Mha) in Turkey is Zn deficient. The high prevalence of Zn-deficient soils in Turkey has been suggested as a major cause of Zn deficiency and to be indirectly related to deficiencies of other micronutrients. 

Lentil is an important dietary source of protein, fiber, minerals, vitamins, and antioxidant compounds and is also an excellent source of macronutrients (P, K, Ca, Mg, and Na), micronutrients (Fe, Zn, Cu, and Mn), and trace elements (Al, Cr, Ni, Pb, Co, Se, Mo). Enrichment of food crops with mineral nutrients is currently a high-priority research area. Producing micronutrient-enriched cultivars (biofortification), particularly those with increased Zn and Fe either agronomically or genetically, and improving the bioavailability of these minerals are considered a promising and cost-effective method to manage micronutrient deficiencies. One approach that can be used to increase the level of mineral nutrients in food crops is to identify natural variants that have favorable traits and use these variants to develop new cultivars. 

There are many lentil landraces owing to differences in traditional farming systems and taste preferences in regions where lentils are cultivated. Agronomists must assess the amount of variability in a trait within the germplasm of a crop to determine whether this trait can be enhanced. South-eastern Turkey is the core area of plant domestication in the Fertile Crescent. This region is located at the junction of the East Mediterranean and Anatolian regions and is thought to be the place where einkorn wheat and various legume species such as lentil, chickpea, field pea, and faba bean were first domesticated [10]. Previous studies showed that lentil germplasm from the Mediterranean area has greater genetic diversity than germplasm from south Asia and the USA [2, 11–13].

Recently, a group of researchers from the Department of Field Crops, University of Cukurova, Adana, and another group from the University of Dicle, Diyarbakır, Turkey, have been evaluating genetic variations in lentil landraces collected from South-Eastern Turkey by analysis of morphological traits and DNA markers. These studies indicated that the Turkish lentil landraces had substantial genetic diversity at the genotypic and phenotypic levels [14, 15]. However, these previous studies did not investigate the mineral compositions of lentil landraces. These landraces are a potential genetic resource for biofortification of lentils with increased micronutrients. In the present study, we examined the genetic variation in macronutrients (P, K, Mg, and Ca), micronutrients (Zn, Fe, Cu, and Mn), protein content, seed size, and seed weight to identify germplasm that could be used to improve the nutritional quality of lentil in Turkey as well as in the Mediterranean region and/or to provide information to international breeder interested in Turkish Genetic resources. 

## 2. Materials and Methods

### 2.1. Plant Material

Thirty-nine Turkish lentil landraces and 7 commercial lentil cultivars were examined in this study. These landraces were collected from nine provinces in southeast Turkey, and all were of the *microsperma *variety. Information about these landraces and cultivars, collection sites, and years of release has been provided previously [14, 15].

### 2.2. Experimental Design and Crop Sowing

 All landraces and cultivars were sown in November 2007 in well-prepared seed beds, using a randomized completely blocked design with three replicates per sample. The field trail was conducted at a research and experimental area of the Seed Science and Technology Department, Vocational School of Kozan, which has an eastern Mediterranean climate. All genotypes were grown in plots of three rows, each 3 m in length, with 10 cm between plants within a row and 45 cm between rows. All plots were treated identically with standard local agricultural practices. Seeds of all lentil landraces and cultivars were harvested on June 15, 2008.

### 2.3. Mineral Nutrient Analysis

Seed samples (0.4 g) were digested in a closed microwave digestion system (MARSxpress, CEM Corp.) in 5 mL of concentrated HNO_3_ and 2 mL of concentrated H_2_O, and then analyzed for mineral nutrients with an inductively coupled plasma optical emission spectrometer (ICP-OES; Vista-Pro Axial; Varian Pty Ltd, Mulgrave, Australia). Nitrogen was measured using AOAC method 997.09 [16] on a Leco TruSpec CN3342 System (Leco Corp., St Joseph, MI, USA) with a 0.2 g sample.

### 2.4. Protein Analysis, Seed Size, 100-Seed Weight

 The protein content of seeds was determined using a standard method [16]. The weight of 100 seeds was obtained from 4 samples of 100 randomly selected seeds from each plot. Seed size was calculated using a micrometer to within ±0.01 mm [17].

### 2.5. Statistical Analysis

Analysis of variance was performed for each trait. Means were compared using Duncan's multiple-range test. Associations among traits were assessed using the Pearson correlation coefficient (*r*). Grouping of landraces based on mineral composition was performed by principal component analysis (PCA). JMP software [18] was used for all statistical analyses.

## 3. Results

Table 1 shows the mean, maximum, and minimum of all analyzed variables in the 39 landraces and 7 cultivars. The landraces and cultivars differed significantly in all observed morphological traits and also had considerable variation in mineral levels (Table 2). Mean seed P content for all landraces was 4.15 g kg^−1^ and ranged from 3.16 to 5.33 g kg^−1^. The Kahmar1, Diy-Haz, and Siirt-Beş landraces had the highest P levels and the Diy-Çın, Şir-Sil2, and Diy-Mer landraces had the lowest P levels. The mean K and Mg concentrations of the landraces were 7.54 g kg^−1^ (range: 6.38–9.50 g kg^−1^) and 1.06 g kg^−1^ (range: 0.89–1.26 g kg^−1^), respectively. The Kahmar1, Mar-Kız, and Şurfa-Siv landraces had the highest K levels and the Mar-Kız2, Diy-Çın, and Sir-Sil2 landraces had the lowest K levels. The Kahmar1, Kahmar2, and Diy-Haz landraces had the highest Mg levels and the Diy-Mer and Diy2 landraces had the lowest Mg levels (Table 2). 

The mean K and Mg concentrations in the 7 lentil cultivars were 7.38 g kg^−1^ (range: 6.71–8.38 g kg^−1^) and 0.99 g kg^−1^ (range: 0.85–1.15 g kg^−1^), respectively. The mean concentration of Ca in the landraces was 0.85 g kg^−1^ (range: 0.48–1.28 g kg^−1^) and the mean concentration of Ca in the cultivars was 0.82 g kg^−1^ (range: 0.69–1.02 g kg^−1^). The Diy-Dic1, Diy-Kulp, and Diy-Dic2 landraces had the highest Ca levels, and the Şır-Sil2, Şır-Sil3, and Diy-sil2 landraces had the lowest Ca levels (Table 2). 

The mean Cu concentrations of landraces and cultivars were 12.10 mg kg^−1^ (range: 9.10–16.92 mg kg^−1^) and 11.73 mg kg^−1^, respectively. The Kahmar1, Diy-Krcd, and Mar-Kız3 landraces had the highest Cu levels, and the Diy-Mer1, Mar-Kız1, and Şır-Sil3 landraces had the lowest Cu levels. 

The amount of Fe in landraces varied from 48.96 to 81.39 mg kg^−1^, and the mean was 63.61 mg kg^−1^. The seven cultivars had a mean Fe concentration of 58.94 mg kg^−1^ and the range was 49.40 to 69.90 mg kg^−1^. The Diy-Kulp, Kahmar1, and Mar-Kız landraces had the highest Fe levels and the Diy-Haz, Gantep-Niz, and Şır-Ciz landraces had the lowest Fe levels. The average Zn concentration of the landraces was 55.01 mg kg^−1^ and the range was 42.30 to 73.10 mg kg^−1^. The Kahmar1, Adıy-kah2, and Diy-Kulp landraces had the highest Zn levels. The mean Mn contents of the landraces and cultivars were 13.43 mg kg^−1^ and 13.49 mg kg^−1^, respectively, and the ranges for all landraces and cultivars were 11.5 to 16.2 and from 11.5 to 15.4 mg kg^−1^. The Kahmar2 and Adıy-Kah2 landraces had the highest Mn levels, and the Diy-Haz and Mar-Kız1 landraces had the lowest Mn levels (Table 2). 

The mean protein content of the landraces was 25.60% and the range was 22.72 to 31.88%. The Şır-Kum, Şurfa-Vir, Kahmar1, and Kahmar2 landraces had the highest protein content, and the Diy-Haz, Diy-Oğl, and Diy2 landraces had the lowest protein content. The 100-seed weight of the Turkish landraces ranged from 1.68 to 4.03 g with a mean of 2.8 g. The Şır-Sil2, Diy-Krcd, and Mar-Kız5 landraces had the greatest 100-seed weight, and the Diy-Kulp, Diy-Dic2, and Batman landraces had the lowest 100-seed weight. The seed size of the landraces ranged from 3.99 to 5.14 mm, with a mean of 4.5 mm (Table 1). 

### 3.1. Seed Mineral Association

Table 3 shows the correlation coefficients among the different mineral contents and other traits in the 39 landraces and 7 cultivars. Correlation analysis indicated numerous significant positive and negative correlations. The large number of observations increased the test power, giving significance to most of the correlations. Hence, only results with *r*-values greater than 0.4 are discussed here. Seed P content was positively correlated with K, Mg, Ca, Cu, Zn (*P* < 0.01 for all), and Fe (*P* < 0.05). K was positively correlated with Cu and Zn (*P* < 0.01 for both). Mg was positively correlated with Cu and Zn (*P* < 0.01). Ca was positively correlated with Zn (*P* < 0.01) but negatively correlated with seed size and 100-seed weight (*P* < 0.05 and 0.01 for both). Cu was positively correlated with Zn, Fe, and seed size (*P* < 0.01, 0.01 and 0.05 resp.). Fe had a strong positive correlation with Mn (*P* < 0.01), Zn (*P* < 0.01), and protein content (*P* < 0.01). Mn was positively correlated with Zn (*r* = 0.40, *P* < 0.01). Zn was negatively correlated with seed size and seed yield (*P* < 0.05 for both). Seed size had a strong positive correlation with 100-seed weight (*r* = 0.71, *P* < 0.01). 

**Figure 1:**
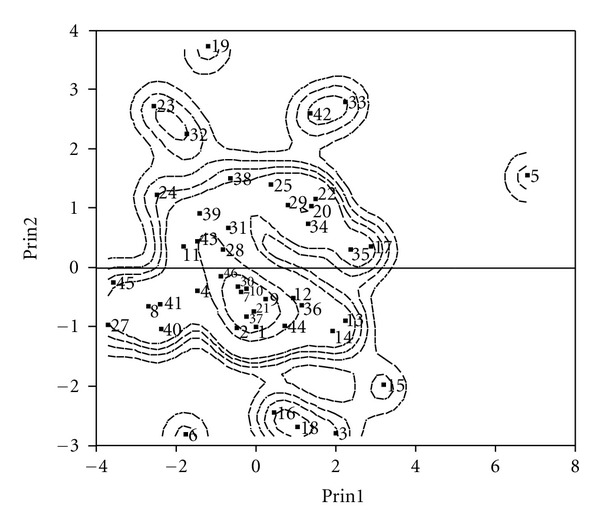
Scatter diagram of the lentil landraces based on studied traits.

Finally, we used PCA to assess the patterns of variations by considering all variables simultaneously. Using PCA based on the correlation matrix, we calculated eigenvalues, percentage of variation, and load coefficients of the first six components for all traits. The first four PCs accounted for 79.45% of the variability (Table 4). PC1 accounted for 36.90% of the total variation, and P, Zn, Mg, and K had the highest positive coefficients. PC2 explained 20.38% of the total variation, and seed size, 100-seed weight, Mn, and Cu had the highest positive coefficients. PC3 accounted for 13% of the total variation, and seed potassium was the main trait. PC4 explained 9% of the variation, and seed protein was the main trait (Table 4). The scattering and relationship of lentil landraces according to principal component analysis are shown in Figure 1.

## 4. Discussion

Providing safe, nutritious, and affordable food is a major challenge faced by developing nations, and more than 170 million preschool children and nursing mothers are adversely affected by micronutrient malnutrition [19]. Micronutrient deficiency will likely continue into the future, given that animal protein is unaffordable in many developing countries [20]. Supplementation of cereal grains with high-protein leguminous seeds is one strategy to improve the diets of people in poor countries [21]. Yadav et al. [22] reported that consumption of seed legumes could play a significant role in reducing the prevalence of nutrient deficiency and malnutrition in diverse populations. Dietary supplementation, fortification, and diversification are traditionally used to reduce micronutrient malnutrition. However, this approach is not feasible in developing countries because of the lack of social and economic infrastructure. Thus, there is an urgent need to develop long-term and sustainable solutions to reduce micronutrient malnutrition in developing countries. Nutritionists have proposed a complementary solution to malnutrition termed “biofortification or genetic improvement” [23]. 

Biofortification and/or plant breeding is a widely accepted strategy and the most sustainable approach that may increase both essential micronutrients concentrations and their bioavailable form in plant foods through genetic improvement. It is also a cost-effective way to minimize the extent of mineral deficiencies, especially deficiencies of micronutrients such as Fe, Zn, Cu, and Ca in economically disadvantaged populations. Thus, new legume varieties with high micro- and macronutrient contents could improve the nutritional status of people in developing countries. On average, global pulse consumption is in decline, but lentil consumption is increasing faster than human population growth, making this species ideal for biofortification. Thavarajah et al. [23] showed that lentil has great potential as a fortifiable crop. Breeding for increased mineral concentrations requires knowledge of natural variations among available germplasm. Landraces provide great potential for improvement of lentil, and their characterization serves as a starting point for studies that aim to improve the micro- and macronutrient contents.

In this study, we determined micro- and macronutrients, protein content, and 100-seed weight in 39 lentil landraces and 7 cultivars. We grew all landraces and cultivars under the same conditions to eliminate the role of environment on observed variations. We found impressive genetic variation in the lentil germplasm for the investigated micro- and macronutrients. The range of Zn concentration of Turkish lentil landraces (42–73 mg kg^−1^; Table 1) was higher than that of Canadian grown lentil (44–54 mg kg^−1^), whereas Fe concentration of Turkish lentil was lower than that of Canadian lentil [23]. This suggests that genotypic variation in lentil landraces provides good opportunities for improvement of cultivated lentil. In addition, genotypes with high micro- and macronutrient levels might be suitable for studying the mechanisms of mineral element accumulation and transport. The mineral characteristics of the crop plants depend on genetic and environmental factors. Variation in the different landraces for mineral characteristics also depend upon the level of soil fertility, soil type, seed characteristics, seed composition, climatic factors, and others. Unconscious selection by local farmers could also have affected lentil diversity in mineral uptake. Local landraces from South-Eastern Turkey are recognized as genetically diverse [14, 15, 24]. Future studies should be conducted under different environmental conditions to better establish the diversity of these landraces.

Lentil landraces had higher average values and ranges of macro- and microelements and other traits than cultivars (Table 2). In particular, the range of Zn and Fe levels in landraces was greater than that of cultivars. The Kahmar1 landrace had the greatest amounts of Zn, Cu, P, K, Mg, and Fe and moderate Ca and Mn concentrations. This landrace also had large seeds and high seed protein content. The Diy-Kulp landrace had high Fe and Ca levels, with moderate Zn and Mn levels. The Mar-Kız landrace had high Fe content, 100-seed weight, and Mn and K contents, and moderate Zn and protein content. The Kahmar2 landrace had the highest Mn level and also had high levels of Cu, Fe, Mg, and protein. The Ady-Kah2 landrace also had high levels of Fe, Mn, Zn, K, and P. These landraces should be considered for use in breeding programs to increase the mineral contents of lentil. Similarly, the Çağıl2004 cultivar had the highest amounts of most minerals and the highest protein; therefore, this cultivar also has great potential for breeding programs. 

We investigated the relationships of lentil traits by correlation analysis and PCA. Several minerals were positively correlated with each other, possibly pointing to common uptake pathways or transporters (Table 3). Interestingly, Zn level had a significant and positive correlation with all other macro- and micronutrients. The positive relationships between Zn and P, and Zn with Mg may be due to the well-known effect of phytate on binding of Zn and Mg in seeds [25]. The positive association of Zn with other minerals demonstrates that selection for high Zn concentration may indirectly select for higher levels of other macro- and micronutrients. Most mineral elements, particularly P, Ca, and Zn, were negatively correlated with 100-seed weight in our landraces and cultivars. Seed weight and seed size are the most important traits associated with crop yield. There has been concern that high seed mineral concentration may result from a “concentration effect” as a consequence of small seed. Thus, it is possible that genotypes that produce low seed yield might have high concentrations of minerals. This inverse relationship of micronutrient concentration and seed size has been documented in other crops [26–28]. The protein content of lentil seed had a significant positive correlation with Mg and Fe, but the correlation with Zn was not significant. Lentil protein content was not significantly associated with seed size and 100-seed weight. The positive association of seed protein content with Fe and Mg levels could be useful for lentil breeders who seek to biofortify lentil seed for high protein and Fe. Correlations between traits can be caused by genetic linkage, pleiotropic, or environmental effects. Environmental effects can force evolution of traits in the same or opposite directions [17]. Thus, the correlations reported here must be seen as provisional until multilocation testing can show the relative contributions of environment and genes.

As discussed above, more than three billion people worldwide have mineral deficiencies. Lentil is an indispensable supplementary food in many countries, particularly in Asia, Middle Eastern countries, and Turkey. In Asia, particularly in Pakistan, India, and Bangladesh, lentil is an integral part of the diet. Red lentil is very popular in Turkey and other Mediterranean countries owing to its abundant nutritional and functional components. Thus, even a small increase in the nutritive value of lentil seed may be highly significant for improvement of human nutrition. 

In summary, we identified considerable variation in the macro- and micronutrient contents of lentil landraces and cultivars. Our results provide a useful foundation for the development of new cultivars of lentil that have high mineral content. In particular, some of the landraces that we studied could be used to develop more nutritional varieties of lentil and reduce mineral element deficiencies in developing countries. Identification of genetic variation is essential for achieving improvements in the mineral content of crops. Such variation can also be used to identify quantitative trait loci (QTL) associated with mineral uptake and transport. We also found significant positive and negative correlations among different traits, suggesting correlation among phenotypic characteristics. The data provided in this study provide an important basis for improvement of lentil, but multi-location trials conducted over several years are needed to more completely evaluate lentil landraces. Lentil landraces from Turkey could be useful for improving the micro- and macronutrient content of lentil seed through genetic improvement.

## Figures and Tables

**Table 1:** Mean and range variation of mineral elements and other traits of Turkish lentil landraces and cultivars.

Parameters	Landraces			Cultivars		
	Mean	Max	Min	Mean	Max	Min
Phosphorous (g kg^−1^)	4.15	5.33	3.16	3.47	3.94	2.86
Potassium (g kg^−1^)	7.54	9.50	6.38	7.38	6.71	8.38
Magnesium (g kg^−1^)	1.060	1.260	0.89	0.99	1.15	0.85
Calcium (g kg^−1^)	0.85	1.28	0.48	0.82	1.02	0.69
Copper (mg kg^−1^)	12.10	16.92	9.10	11.73	14.50	9.50
Iron (mg kg^−1^)	63.61	81.39	48.96	58.94	69.90	49.40
Manganese (mg kg^−1^)	13.43	16.20	11.50	13.49	15.40	11.50
Zinc (mg kg^−1^)	55.01	73.10	42.30	53.49	61.30	46.90
Protein contents (%)	25.60	31.88	22.72	24.43	26.54	23.60
Seed size (mm)	4.50	5.14	3.99	4.6	4.60	4.06
100 seed weight (g)	2.80	4.03	1.68	3.02	3.33	2.58

**Table 2:** Mean values of micro- and macronutrients, protein contents, seed size, and hundred-seed weight of lentil landraces.

Landraces/cultivars	No. in fig.	P^†^	K^†^	Mg^†^	Ca^†^	Cu^‡^	Fe^‡^	Mn^‡^	Zn^‡^	Protein (%)	SS (mm)	HSW (g)
Diy-Haz	1	5.33 b	8.11 b–e	1.18 a–c	0.76 n–s	12.3 d–j	49.0 q	10.5 q	52.3 l–r	22.72 r	4.36 h–k	3.35 b–g
Diy-Oğl	2	4.22 f–l	7.24 j–s	0.97 p–t	0.92 e–l	12.4 d–j	63.1 e–m	12.9 g–p	54.6 h–n	23.48 q	4.17 m–r	2.73 h–l
Diy-Kulp	15	4.71 c–g	7.37 h–p	1.11 c–I	1.22 a-b	11.8 e–m	81.4 a	13.8 c–l	64.2 b-c	28.48 d-f	3.99 s-t	1.68 o
Diy-Dic1	16	4.24 f–l	6.65 s–v	1.05 f–p	1.28 a	11.4 g–m	59.9 h–o	12.6 i–p	58.4 d–k	26.59 i-j	4.10 p–t	2.22 l–o
Diy-Dic2	18	4.46 d–j	6.99 n–v	1.06 e–n	1.18 a-b	10.5 k–o	59.9 h–o	12.9 g–p	59.2 c–h	30.29 b	4.06 q–t	1.87 n-o
Diy-Krcd	19	3.51 o–r	7.35 h–p	1.00 i–s	0.77 m–r	14.3 b-c	69.2 b–e	14.7 a–f	43.7 t-u	27.40 g-h	5.14 a	3.78 a-b
Diy-Mer	27	3.20 r-s	7.06 m–u	0.89 t-u	0.55 u–w	9.1 o	55.0 n–q	12.9 g–p	45.4 s–u	24.47 p	4.23 k–q	2.95 e–j
Diy-Sil2	28	3.95 i–o	6.92 p–v	1.05 g–q	0.52 u–w	12.0 e–l	67.6 b–h	13.6 d–m	47.5 p–u	28.20 d–f	4.46 f–i	2.34 k–n
Diy-Sil1	20	3.92 k–p	7.99 b–g	0.99 m–s	0.88 g–n	13.2 b–f	74.3 a–c	15.3 a–c	63.1 b–e	27.22 h	4.23 k–q	3.00 e–j
Diy-Erg	34	4.30 e–k	8.03 b–e	1.16 b–d	1.12 b–d	11.7 e–m	65.7 e–k	14.5 b–g	56.7 g–l	26.49 i	4.54 d–g	3.33 b–g
Diy-Çın	8	3.16 r-s	6.45 u–v	0.97 p–t	0.77 m–r	10.8 i–n	57.9 l–p	12.8 h–p	43.6 t-u	28.98 d	4.16 n–r	3.03 d–j
Diy1	38	3.69 m–r	7.64 e–m	1.10 c–j	0.73 o–t	12.7 c–h	59.2 j–o	12.3 j–p	54.8 h–n	27.80 d–f	4.70 c-d	3.63 a–d
Diy2	39	3.61 n–r	7.40 f–p	0.94 s-t	0.81 l–q	11.7 e–m	63.8 e–l	14.0 c–j	55.0 h–m	24.08 p	4.47 f–i	3.53 a–e
Batman	3	4.98 b–d	8.28 b–d	1.17 b–d	1.14 b-c	12.2 e–j	57.7 l–p	12.0 m–q	51.2 l–r	28.21 e–g	3.99 s-t	1.86 n-o
Kahmar1	5	6.45 a	9.50 a	1.26 a	1.04 c–e	16.9 a	78.5 a	13.4 e–o	73.1 a	30.44 b	4.54 e–g	2.77 g–l
Kahmar2	33	4.19 g–m	7.96 b–h	1.20 a-b	0.93 e–l	14.0 b–d	67.0 c–i	16.2 a	58.1 e–k	30.54 b	4.81 b-c	3.18 c–i
Adıy-Kah	7	3.93 k–p	7.24 j–s	0.97 p–t	0.98 e–j	11.5 g–m	66.5 d–j	13.4 e–o	51.9 l–r	28.78 d–f	4.31 i–n	2.80 g–l
Adıy-Kah2	17	4.72 c–f	8.29 b–d	1.14 b–f	0.96 e–k	12.5 d–I	68.4 b–f	16.0 a-b	66.0 b	25.97 k–n	4.42 g–j	2.31 k–n
Gantep	4	4.09 i–n	7.60 e–n	0.99 m–s	0.85 j–o	12.1 e–k	53.8 n–q	12.2 l–p	47.2 q–u	24.65 p	4.54 d–g	2.94 e–j
Gantep-Niz	26	3.59 n–r	7.92 b–I	1.01 j–s	0.88 g–n	12.4 d–j	50.7 p-q	13.9 c–k	51.2 l–r	25.45 m–o	4.52 f–h	3.45 a–f
Mar-Kız2	11	3.52 o–r	6.38 v	0.99 m–s	0.65 r–u	12.1 e–k	60.2 g–o	12.9 g–p	50.2 m–s	29.65 c	4.33 i–m	3.11 d–j
Mar-Kız3	29	4.48 d–i	7.38 g–p	1.01 k–s	0.76 n–s	14.5 b	65.9 e–k	13.6 d–n	61.1 b–g	26.25 j–m	4.53 f-g	2.88 f–k
Mar-Kız4	30	3.87 k–q	8.01 b–f	1.03 i–r	0.54 u–w	12.6 c–h	61.1 f–n	12.2 k–p	54.8 h–n	26.39 i-j	4.13 o–t	2.81 g–l
Mar-Kız	35	4.30 e–l	9.07 a	1.12 b–h	0.84 k–p	12.3 d–j	74.9 a-b	15.3 a–c	58.1 e–k	26.17 i–l	4.15 n–s	2.58 j–m
Mar-Kız5	32	3.48 o–r	6.950 v	1.07 e–n	0.76 n–s	12.1 e–k	55.8 m–q	14.1 c–i	48.2 o–t	28.25 d–e	4.92 b	3.72 a–c
Mar-Kız1	6	4.18 h–m	7.30 i–r	0.98 n–s	0.93 e–l	9.4 n-o	53.5 n–q	11.5 p-q	42.3 u	28.19 d–g	4.11 p–t	2.29 k–n
Mar-Om	12	4.79 c–e	7.73 d–k	1.09 c–k	0.82 l–p	12.5 d–h	66.5 d–j	12.0 n–q	56.2 g–l	28.09 f–h	4.16 m–r	3.02 e–j
Siirt-Beş	13	5.20 b-c	8.41 b	1.20 a-b	0.89 f–m	11.8 e–m	58.6 k–o	13.4 e–o	61.5 b–g	26.43 i-j	4.24 k–p	2.58 j–m
Şurfa-Har	9	4.24 f–l	7.55 e–o	1.03 i–r	0.64 s–v	11.6 f–m	74.3 a–c	12.2 l–p	55.9 g–l	28.02 d–f	4.13 o–t	2.68 i–l
Şurfa-Hil	10	3.91 k–q	6.99 n–v	1.0 k–s	0.86 h–n	12.0 e–l	67.8 b–g	13.4 e–o	56.2 g–l	26.92 i	4.20 k–r	2.79 g–l
Şurfa-Vir	25	3.91 k–q	7.05 m–u	1.09 d–I	0.81 l–q	13.1 b–g	64.3 e–l	13.9 c–k	56.8 f–l	31.13 a	4.54 d–g	3.19 b–i
Şurfa-Sur	31	3.77 l–q	7.00 n–v	1.04 h–q	0.61 t–v	12.3 d–j	57.8 l–p	13.1 f–p	58.5 c–j	29.48 c	4.42 g–j	3.08 d–j
Şurfa-Siv	36	4.30 e–k	8.43 b	1.15 b–e	0.82 l–p	13.0 b–g	60.7 f–o	13.1 f–p	52.9 j–p	26.70 i–k	4.05 r–t	2.71 i–l
Şır-Kum	14	4.69 c–h	7.08 l–t	1.07 e–m	0.77 m–r	10.4 l–o	74.0 a–d	14.9 a–e	64.1 b–f	31.88 a	3.99 t	2.00 m–o
Şır-İdil	21	3.98 i–o	7.84 b–j	1.02 j–s	0.98 h–n	11.2 h–m	69.3 b–e	12.9 g–p	52.7 k–p	24.75 p	4.34 i–l	2.69 i–l
Şır-Sil1	22	4.24 f–l	7.89 b–I	1.13 b–g	0.99 e–h	13.4 b–e	59.6 i–o	13.1 f–p	63.9 b–d	28.34 d	4.90 b	2.87 f–k
Şır-Sil2	23	3.17 r-s	6.69 r–v	1.02 j–s	0.48 w	10.4 l–o	69.5 b–e	14.8 a–f	47.1 q–u	28.14 d–f	4.70 c–e	4.03 a
Şır-Sil3	24	3.41 p–r	6.47 t–v	1.01 k–s	0.51 u-v	10.3 m–o	65.2 e–l	13.3 f–o	49.1 n–t	27.51 h	4.77 b-c	3.14 c–j
Şır-Ciz	37	4.10 i–n	7.69 d–l	0.97 o–t	1.00 d–g	11.5 g–m	53.3 o–q	14.2 c–i	58.5 c–j	25.37 o	4.26 j–p	2.77 g–l

Varieties												
Fırat 87		3.82 k–q	7.56 e–o	0.98 n–s	0.72 p–t	10.4 l–o	49.4 q	11.5 p-q	46.9 r–u	25.88 l–n	4.47 f–i	2.88 f–k
Yerli Kır.		3.18 r-s	6.66 s–v	0.96 q–t	0.77 m–r	12.5 d–h	54.2 n–q	11.9 o–q	48.2 o–t	26.38 i–l	4.34 i–l	2.77 g–l
Çağıl 2004		3.94 j–o	8.38 b-c	1.06 f–o	0.69 q–t	14.5 b	69.9 b-e	15.4 a–c	61.3 b–g	26.54 i-j	4.57 d–g	3.33 b–h
Çiftçi		3.20 r-s	6.71 q–v	0.97 p–t	0.86 i–n	12.5 d–h	61.0 f–n	14.5 b–g	53.3 i–o	25.46 o	4.28 j–o	3.12 c–j
Kafkas		3.91 k–q	7.78 c–k	1.15 b–e	0.87 b–e	10.7 j–o	63.5 e-m	14.2 c–i	58.7 c–i	25.39 m–o	4.06	2.58 j–m
Emre		2.86 s	7.33 i–q	0.85 u	0.81 l–q	9.5 n-o	54.4 n–q	12.7 i–p	50.0 m–s	23.61 q	4.60 d–f	3.23 b–i
Özbek		3.39 q–s	7.21 k–s	0.95 r–t	1.02 c–f	12.0 e–m	60.3 g–o	14.3 c–h	56.1 g–l	25.81 n-o	4.18 l–r	3.16 c–j
*Mean square *		0.53**	0.56**	0.01**	0.03**	2.91**	68.5**	1.84**	50.8**	4.67**	0.08**	0.26**

*P < 0.05, **P < 0.01, means sharing similar letter are statistically not different.

^†^g kg^−1^.

^‡^mg kg^−1^.

SS: seed size, HSW: g 100 seeds^−1^.

**Table 3:** Correlation coefficients between seed macro- and microelement concentrations among lentil landraces and cultivars.

	K	Mg	Ca	Cu	Fe	Mn	Zn	Protein	Seed size	HSW
P	0.67**	0.76**	0.44**	0.44**	0.30*	−0.09	0.63**	0.20	−0.25	−0.47**
K		0.63	0.26	0.50**	0.27	0.15	0.53**	−0.13	−0.03	−0.13
Mg			0.30*	0.48**	0.30*	0.15	0.54**	0.34**	0.02	−0.21
Ca				0.11	0.10	0.07	0.43**	0.03	−0.30*	−0.50**
Cu					0.37**	0.27	0.50**	0.19	0.33*	0.18
Fe						0.57**	0.53**	0.38**	−0.05	−0.17
Mn							0.40**	0.19	0.24	0.14
Zn								0.23	−0.16*	−0.33*
Protein									0.05	−0.19
Seed size										0.71**

*P < 0.05, **P < 0.01.

**Table 4:** Eigenvectors, eigenvalues, individual and cumulative percentages of variation explained by the first four principal components (PC) of 39 Turkish lentil landraces and 7 cultivars.

Variables	Eigenvectors			
	PC1	PC2	PC3	PC4
P (g kg^−1^)	0.42314	−0.15612	0.24258	0.19210
K (g kg^−1^)	0.35888	0.04938	0.40728	−0.22127
Mg (g kg^−1^)	0.39849	0.05795	0.20082	0.30920
Ca (g kg^−1^)	0.26304	−0.28293	−0.01273	−0.22216
Cu (mg kg^−1^)	0.30134	0.35831	0.20633	0.07484
Fe (mg kg^−1^)	0.29464	0.20119	−0.44493	−0.16168
Mn (mg kg^−1^)	0.16310	0.37360	−0.41613	−0.43299
Zn (mg kg^−1^)	0.41977	0.03678	−0.09768	−0.19881
Protein (%)	0.16797	0.10679	−0.46767	0.69840
SS (mm)	−0.09087	0.55164	0.18896	0.14548
HSW (g)	−0.21742	0.51408	0.23902	−0.03872

Eigenvalue	4.0595	2.2427	1.4401	0.9968
Percent	36.9049	20.3879	13.0920	9.0621
Cum. percent	36.9049	57.2927	70.3847	79.4468
